# Training of neurologists for the 21st century: cultural and professional skills

**DOI:** 10.1590/0004-282X-ANP-2022-S104

**Published:** 2022-08-12

**Authors:** Ricardo Nitrini

**Affiliations:** 1 Universidade de São Paulo, Hospital das Clínicas, Faculdade de Medicina, Departamento de Neurologia, São Paulo SP, Brazil.

**Keywords:** Forecasting, Neurologists, Teaching, Mentoring, History, 21st Century, Humanism, Clinical-Anatomic Correlation, Previsões, Neurologistas, Ensino, Tutoria, História, Século XXI, Humanismo, Correlação Anátomo-Clínica

## Abstract

Training of neurologists for the near future is a challenge due to the likely advances in neuroscientific methods, which will change much of our knowledge on diagnosis and treatment of neurological diseases. Objective: to comment on what may be more likely to be a constant in the very near future and to recommend how to prepare the neurologist for the 21st century. Methods: through a critical review of recent articles on the teaching of Neurology, to present a personal view on the subject. Results: Diagnostic methods and therapeutic resources in Neurology will be greatly improved, but the central core of teaching young neurologists will continue to be the clinical/anatomical correlation. The neurologist must be prepared to be the primary physician in the care of patients with neurological disorders, although the roles of consultant and clinical neuroscientist must also be considered. In addition to technical knowledge, the neurologist must be prepared to discuss not only distressing issues related to the specialty, such as the risks of genetic diseases for family members of their patients, the inexorable progression of some diseases and the need for palliative care, but also problems not directly related to Neurology that cause anxiety and depression in the patient or that are the main reason for the initial consultation. Conclusion: neurology will be an even more important area of medicine and the neurologist must be well prepared to be the primary doctor to diagnose, treat and follow the patient with neurological disorders. In addition to technical knowledge, training in doctor-patient relations should be highlighted.

## Resumo

A formação do neurologista para o futuro próximo é um desafio devido aos prováveis avanços nos métodos da neurociência, que mudarão muito do nosso conhecimento sobre diagnóstico e tratamento de doenças neurológicas. Objetivo: comentar o que pode ser mais constante no futuro próximo e propor como preparar o neurologista para o século XXI. Métodos: por meio de uma revisão crítica de artigos recentes sobre o ensino da Neurologia, apresentar uma visão pessoal sobre o assunto. Resultados: Os métodos diagnósticos e os recursos terapêuticos em Neurologia serão muito aprimorados, mas o núcleo central do ensino de jovens neurologistas continuará sendo a correlação clínico-anatômica. O neurologista deve estar preparado para ser o médico principal no atendimento de pacientes com distúrbios neurológicos, embora os papéis de consultor e neurocientista clínico também devam ser considerados. Além do conhecimento técnico, o neurologista deve estar preparado para discutir não apenas questões angustiantes relacionadas à especialidade, como os riscos de doenças genéticas para os familiares de seus pacientes, a progressão inexorável de algumas doenças e a indicação de cuidados paliativos, mas também problemas não diretamente relacionados à Neurologia que causam ansiedade e depressão no paciente ou que são a principal causa da consulta. Conclusão: a neurologia será uma área ainda mais importante da medicina e o neurologista deve estar bem-preparado para ser o médico principal para diagnosticar, tratar e acompanhar o paciente com distúrbios neurológicos. Além do conhecimento técnico, a formação humanística deve ter destaque.

## INTRODUCTION

 The training of neurologist for the future is a challenge due to discoveries that certainly will change much of our knowledge on diagnosis and treatment of neurological diseases. Predicting what will happen in the future in any area of medical knowledge is practically impossible, because “progress largely depends on discovery, and discovery cannot be predicted”^1^. In its March 1949 edition, Popular Mechanics, a well-known magazine, predicted: *Where a calculator like the ENIAC today is equipped with 18,000 vacuum tubes and weighs 30 tons, computers in the future may have only 1,000 vacuum tubes and perhaps weigh only 1½ tons* ^*2*^ *.*

 The best neurologists of the early 1970s, when I started my residency in neurology, could not predict the impact on neurology of information technology, neuroimaging techniques and genetics.

 In this sense, our forerunners were much more conservative than we are now when thinking about the very near future. We are now seeing so many changes in just a few months due to technology that it is common to hear that “we just don’t know where these new machines (mainly related to information technology) are going to take us”.

 Although it is not easy to understand that in the next 50 years we can make progress as great as was achieved in the previous 50 years, most likely the advance will be even greater, as it has been over time. How to prepare the neurologist for these new times? This is a huge challenge, but as a starting point we can use the fact that we must work with a less broad perspective, that we will have to prepare the neurologist for the very near future and not for the more distant, as this is almost completely intangible.

 In 2015, Professor Vladimir Hachinski organized a session at the World Congress of Neurology to be held in Santiago, Chile, and invited Morris Freedman and me to share with him the themes he proposed. For me, the suggested theme was: *Constants in a changing world* (what should remain in the neurology of the future). This perspective is also of interest for this presentation and is the one I shall use the most -

always thinking about the very near future. The Department of Neurology of the Medical School of the University of São Paulo, located at Hospital das Clínicas (DN-USP), where I did my residency in neurology and spent my entire academic career will always be my model for criticism and proposals.

 According to Abraham Lincoln, “The best way to predict the future is to create it”^3^. Or as Hachinski (2002)1 wrote: “However, understanding from where we are coming may help guide where we are going”. This is the task that will fall to all those who participate in the training of new neurologists.

Within our limitations, we can think about what kind of doctor the neurologist of the future should be. Which qualifications should neurologists have? And from there, we can imagine a broad, but at the same time flexible curriculum.

## THE IMPORTANCE OF NEUROLOGY IN THE FUTURE

 Neurological diseases are the world's largest cause of disability-adjusted life years (DALYs), or years of a healthy life that are lost to due to death or disability. Neurological disorders caused 9.4 million deaths in 2015, up 36.7 percent from 1990, making them the second leading cause of mortality across the world after cardiovascular disorders^4^.

 Due to the aging of the world population, advances in diagnosis due to neuroimaging, genomics, increasing knowledge in neurosciences and far more available treatments for neurological diseases, neurology is going to be a much more important area of medicine than it is today.

 In 2021, the American Academy of Neurology stated that there were 31 neurology fellowship areas, of which 17 are already accredited while 14 are not yet accredited^5^. Neurology is a large area of medicine with many sub-specialties yet to be developed. But there is a central nucleus for the neurologist's initial training, which is common to all who are to dedicate themselves to each of these sub-specialties.

## PROFESSIONAL SKILLS

 The central core of the training of a neurologist has been that of topographic diagnosis: the clinical/anatomical correlation. Clinical diagnosis in neurology requires several steps: 1) Recognition of impaired function; 2) Identification of what site or sites of the nervous system has been affected (localization); 3) Differential diagnosis of the cause; 4) Use of ancillary testing to diagnose the disease^6^.

 But neurology needs to go far beyond diagnosis, and it has. In this sense, it is worth discussing what role is expected of the neurologist in medical care in general.

## A CONSULTANT FOR DIFFICULT CASES WITH NEUROLOGICAL DISORDERS?

 Should the future neurologist be prepared to be a consultant, who will help other specialists in the diagnosis of neurological diseases and the neurological complications of systemic diseases? With the advancement of neuroimaging techniques, diagnostic methods with biomarkers in cerebrospinal fluid (CSF) and plasma, with genetic diagnostic techniques, it will be very difficult for the non-neurologist to analyze and assess the importance of all the data acquired from a patient. A neurologist will be needed to verify the importance of a neuroimaging finding, a concentration of a biomarker or a mutation of uncertain value. We can imagine that this role of the neurologist as a consultant will grow. But this must not be the only or the most important role of the neurologist of the future^7^.

### Comprehensive Care: the neurologist as the primary physician to diagnose, treat and follow up the patient with neurological disorders?

 There are several reasons for supporting the understanding that the neurologist should be the primary physician in neurological diseases. This may take place as an office neurologist in outpatient clinics or in hospitals, including intensive care units. To be the primary physician the neurologist should also receive training in clinical medicine and psychiatry during the residency program^7^. 

 There is in addition another role, which is going to be shown later: the neurologist as clinical neuroscientist.

## PREPARING THE FUTURE NEUROLOGIST

This session should answer three main questions: Who should teach them? What should they learn? How to teach them?

### Who should teach?

During the residency program, neurology should be learned mainly from neurologists. In the DN-USP, we believe that young former residents (preceptors) are the best for teaching neurology for residents (this has been the method of teaching neurology in our department for more than 50 years). The preceptor is selected among the residents who have just completed the residency program, primarily by their peers, who should evaluate qualities of knowledge, didactics, leadership, and ease of interpersonal relationships. The choice is usually confirmed by the heads of the service.

We currently have two preceptors who are the main trainers of new neurologists, teaching how to take the medical history and perform the neurological examination at the bedside, and discussing cases in small groups. Each year, new preceptors replace the previous ones and try to do their best to be even better than those who preceded them. This method, although old, works very well and should be implemented using online meetings. There are also rounds with the members of the clinical neurologist’s staff. Lectures, rounds and meetings with the clinical neurologist’s staff and other important teachers: neuroimaging specialists, neurophysiologists and neuropathologists. In the future, hybrid meetings, partially face-to-face and partially online will predominate.

### What should the future neurologists learn?

In the teaching of neurology, the initial step is the learning of neuroanatomy. Without this knowledge, it is not possible to progress in the specialty. 

Neuroanatomy in the context of neurology is the best way to teach and to win this challenge. That is why neurologists may be very good teachers of neuroanatomy for future neurologists. When an anatomical structure is linked to a function or to a sign caused by its lesion, the learning of neuroanatomy becomes much more interesting and easier for the future neurologist 

 There are new methods and, for sure, further new methods will be available. Students who took the course with 3D computer-based learning of neuroanatomy achieved better results than those who took the course in an anatomy laboratory^8^. A neuroanatomy teaching method using a smartphone proved to be more efficient than a study carried out with books on anatomy^9^. 

 Correlation with neuroimaging methods and access to an anatomy laboratory will continue to be important. 

### Learning how to take the history

 Neurological assessment begins with a very detailed history of all symptoms and signs and their chronology and is usually what contributes most to a diagnosis. According to Jerome Posner (2013), “Different from all other medical specialties, save perhaps psychiatry, the neurologist is heavily dependent on listening to and interpreting what the patient tells us… If you don’t know what is happening by the time you get to the feet you are in real trouble” (quoted by Nichol & Appleton, 2015)^10^.

 Earlier neurologists took it for granted that “If at the end of taking the history, you do not have a likely diagnosis, take the history again”^11^. 

 The history remains the most important part of the diagnosis and will likely continue to be. Patients often want us to use examinations and even get anxious when we want to know more about the symptoms, when and how they occur and if they interfere with everyday activities. In addition to helping us a lot in diagnosis, history establishes the first human bond between doctor and patient. We learn a little about who our patient is, what he does, what he likes, and the more we can do to get to know him, the better for the future treatment. This is the first P of the three Ps of medicine that we want for the future: **P**ersonalized medicine. Others are **P**reventive and **P**redictive medicine, to which **P**articipatory and **P**urpose-driven medicine were later introduced^12^. 

### Neurological examination

 Neurological examination should always be taught with the clear purpose of allowing clinical/topographic correlation or to allow phenomenological interpretation of the signs, as in the examination of a patient with aphasia or in Balint's syndrome. As Aminoff wrote, “It is up to the present generation of neurologists to ensure by their teaching and example that the skills of the neurological examination are passed intact to their successors”^13^. However, the neurological examination with technical emphasis in the search for a sign must allow time for access to other methods of clinical/topographic correlation. Does it still have the same value as in the past (and is it going to have the same value in the very near future?) Does it need to be taught in all its details as the most conservative aspiration? Does a complete neurological examination need to be performed on all patients? The answer is “no” to all three questions. 

 But the neurological examination will continue to be very important. In many conditions, such as movement disorders, vertigo, and distinguishing between signs caused by neurological diseases or psychogenic (or functional) disorders or malingering is critical. It allows clarification as to which ancillary tests are most needed, greatly reducing the cost of medicine. It is also of great importance to define whether a finding of a neuroimaging or some other test, such as a genetic study, should be valued. This is the best and most efficient method for evaluating the evolution of most neurological diseases with treatment^13^ ^-^ ^15^.

 There will not always be patients with all the important signs we would like to teach. Face-to-face examination of patients is essential. The ability to elicit signals can only be learned by examining. Films, videos and other similar resources should be used and, whenever possible, resident training services should have their own resources, made available through publications or sharing and should also use items available in digital databases, always with care to verify the source and accuracy of the information^16^.

 Even a relatively short neurological examination, in which balance, gait, muscle strength, coordination, reflexes, ocular, facial and bulbar motricity are evaluated is able to establish a closer relationship between the neurologist and the patient. At the end of the neurological examination, many neurologists must have heard: “*Doctor, I have never been so well examined in my whole life”*

 This demonstration of respect for the physician is a very important achievement, which will be reinforced at each new follow-up visit. In addition to the knowledge of neurological diseases and their treatment, the ability to follow the evolution of signs and symptoms, the neurological examination can make the bond with the patient even stronger, and this starts when taking the history. This is another important reason why we should train the neurologist to be the main doctor in diseases of the nervous system, and not simply a consultant^7^ ^,^ ^13^.

### Clinical/topographic correlation (localization of the lesion or dysfunction)

This is the essential knowledge that history, neurological examination and ancillary examinations seek. Establishing the cause of the disease in neurology (the etiological or nosological diagnosis) before knowing the topographic diagnosis would be equivalent to making a diagnosis in clinical medicine without, for instance, assessing whether the disease is affecting the kidneys, liver or heart. Imaging tests, particularly MRI, are very helpful in topographic diagnosis, but there is a need to establish a correlation between the neuroimaging findings and the symptoms and signs. It is common to observe misconceptions in which multiple small areas of hypersignal on MRI in the FLAIR acquisition, or signs of cortical atrophy typical of aging are correlated with signs and symptoms of dementia, for example, or signs of degeneration on spinal MRI with unrelated symptoms. Even more frequent is the misinterpretation of a correlation with other complementary tests such as electroencephalogram and electroneuromyography. Ancillary tests should be requested with prior knowledge of what we are looking for.

The teaching of clinical/topographic correlation is one of the most interesting to the teacher because students of neurology know that they need to master this knowledge. Case studies probably will continue to be the best method of teaching in the emergency unit, wards or outpatient clinics. This should be complemented with group clinical case discussions, in the classroom, with the presence of a senior resident, a preceptor or a member of the clinical staff.

### Nosological or etiological diagnosis

Once the topographic diagnosis is established, the steps that lead to the etiological or nosological diagnosis are similar to those used in medicine in general, although there are specificities in neurology, with tests that are carried out less frequently in other specialties. This learning is mainly acquired in the care of patients in wards and outpatient clinics of various neurological subspecialties. 

Until the advent of neuroimaging, we had the history (anamnesis) and neurological examination as our main weapons for diagnosis. Neuroimaging has largely superseded the power of history and neurological examination for establishing the topographic diagnosis in many conditions such as stroke, neuro-oncology and multiple sclerosis. However in others, such as headache and epilepsy, the history remains the most important item, while in others, such as movement disorders, a neurological examination is essential. The diagnosis in the very near future will continue to depend heavily on the association between clinical history, neurological examination and complementary tests, particularly neuroimaging. 

 Besides the ancillary tests that are common to other specialties, neurologists need to learn how to perform and analyze neuroimaging, electroencephalography, electroneuromyography, CSF and ultrasound for neurovascular diseases. Currently and in the very near future, neurologists should learn how to ask for and analyze liquid biopsies (biomarkers, transcriptomics, proteomics)^17^, results of genomics^17^, and when and how to use artificial intelligence^18^. They should also learn to be aware of advances in neuroscience and its clinical applications, and of advances in information technology (new apps, new programs).

## THE NEUROLOGIST AS A NEUROSCIENTIST

When I started my residency in Neurology, 50 years ago, many of the diseases that we currently diagnose were completely unknown among neurologists. And how did they diagnose conditions such as anti-NMDAr encephalitis, semantic dementia, Lewy body dementia, corticobasal degeneration, inclusion body myositis, and many others? They diagnosed these as other diseases that were already known such as viral encephalitis, Pick’s disease, senile dementia associated with Parkinson’s disease, Parkinson-plus, polymyositis, and so on.

According to Luria (1973)^19^ lesion (disease) creates a window through which we may understand how the brain works. Or, as Miller-Fischer said, “We learn neurology stroke by stroke” (quoted by Louis Caplan, 2020)^20^.

In recent years, the number of newly-described encephalitis cases that were reclassified after identification of genetic mutations due to clinical characterization associated with neuroscience methods or diseases have been very high. 

How were these “new” diseases or new signs for diagnosing other diseases discovered? In many of them, the role of the clinical neurologist was the most important. It is relevant to think that there are many “new” diseases to be discovered and treated. A well-prepared neurologist will be able to recognize and publish a special case report and then a case series. It will be easier if it is associated with neuroimaging, biomarkers, genomics or other neuroscience methods. Working together with other neuroscientists is essential, a possibility increased by the advance of online communication following COVID-19 pandemics^21^. 

In the near future several new devices and neuroscience methods will be available to improve diagnosis of old and new diseases. Considering the probable advances in neuroimaging, we may dream of good portable neuroimaging devices, neuroimaging methods that are less oppressive, without ferromagnetism, methods that will (almost?) reach the refinement of the neuropathological examination (identification of acute or chronic inflammatory reactions, identification of tumor histopathology), more neuroimaging technologies for identifying abnormally processed proteins^17^.

In the last few decades, the most important discoveries that have had an impact on diagnosis and treatment of neurological diseases have been neuroimaging and neuroimmunology, and more recently neurogenetics. The great challenge is the treatment of neurodegenerative diseases. Prevention based on genomics may be the most important discovery (as in SMA). Finally, regeneration of the nervous system seems to be a dream too far for the very near future.

### How to teach them?

Medical residency has the characteristics of “on-the-job learning”, and so it mainly uses participatory teaching methods. Preceptors, who are the main trainers of new neurologists in our department, teach neurological examination at the bedside and discuss cases in small groups. We use case-based sessions focusing on clinical reasoning and diagnostic skills, delivered to small groups, with facilitators who are skilled and experienced in supervising team-work. With COVID-19 pandemics we have learned to participate in online discussions with people from different centers (webinars, meetings, collaborative study groups…)^21^. In addition we are now trying to include telemedicine in the curriculum^22^ as well as the opportunity to learn and contribute to the development of digital neurological examination^23^.

In recent years, the value of each of the methods of teaching has been evaluated and information has been widely disseminated showing that the lecture, or master class, which has been used so much in the teaching of medicine, is one of the least efficient. According to the so-called Learning Pyramid, lectures were efficient only for the learning of the lecturer, but not for the students^24^. 

This information went in the opposite direction to the method most used in many courses, such as the Annual Meetings of the American Academy of Neurology and the meeting we have been organizing since 1989, named Conducts in Neurology^25^. In both, lectures or classes are the main teaching method. In both, however, there is an important difference from the usual conferences: in addition to the lecture, there is a syllabus or a book in which the lecture or lecturer is presented in order to complement and maintain the information. Likewise, notes taken during the conference can make a big difference.

The Learning Pyramid showed average information retention rates obtained with different teaching methods. Current criticisms of the Learning Pyramid make it clear that, in the first place, there has not been a true study that has reached these conclusions and that there is no method that is always superior to the others, while each one of them can be very efficient depending on the context. And finally, they point to the teacher's role in deciding which is the most appropriate method for that learning and its retention^24^.

### After the diagnosis

Despite everything we mentioned about the importance and difficulties in reaching a nosological diagnosis, this set of procedures follows logical steps, like those of an algorithm, which may allow for the replacement of the neurologist by artificial intelligence methods. Or rather, the neurologist can be helped by artificial intelligence, which will expand the training of all neurologists in reaching the correct diagnosis^18^. In the future, a professional with much less knowledge of neurology will be able to achieve more accurate diagnoses using relatively simple data acquisition and artificial intelligence.

During the residency program it is essential that residents develop the ability to follow up their patients. After the diagnosis, informing the patient of the diagnosis, with information about the prognosis and changes in life that will be necessary, the therapeutic approaches and their constant adaptations will require the personal involvement of the professional, which also demands training in doctor-patient attitudes.

At the forefront of the case is the patient, the human being. Empathy, understanding what the patient and his family feel and how to reassure them, how to convey to them the knowledge that the best treatment is being given, even when the prognosis is unfavorable, is one of the most complex tasks for the doctor who deals with serious illnesses, which the neurologist usually does. Empathy, the desire to know the patient, is innate in many doctors who seek medicine out of a desire, often unspoken, to help others. 

But many doctors have a more technical view of professional practice and imagine that pharmacological or surgical therapeutic methods are the ones that really work. They think about the case rather than about the patient. All residents must receive technical training, but we must provide training in the doctor-patient relationship, with discussion as to which difficulties of each patient are also discussed. 

For example, the patient who lives alone suffers a huge impact from some treatments and the follow-up may be impaired; the presence of depression, anxiety, alcoholism, unemployment, drug addiction, among many other conditions, requires Personalized Neurology. The human characteristic of medicine is its most important property.

In many regions of the world, people with personal, family or professional problems look to a religious leader to talk to and get help from in making the right decisions. Today, and probably even more so in the very near future, fewer and fewer people will turn to religious leaders and more and more will turn to doctors for help.

The neurologist must be prepared to discuss not only the distressing issues related to the specialty such as the risks to family members of their patients with genetic diseases, the inexorable progression of some diseases, advance directives of wills, the indication of palliative care, but also problems not directly related to Neurology, but that cause anxiety, depression and anguish in the patient or that are the main reason for the consultation.

The case discussion method can be one of the best, especially in outpatient clinics. Teachers should be concerned with discussing and teaching these skills, particularly those teachers with experience and who recognize that these are the most difficult and also most needed for the good practice of neurology.

In addition to case discussions, training in the doctor-patient relationship is fundamental. The neurologist will not have had time to acquire all the life experiences necessary for the proper understanding of the concerns of his patients. But he may have learned a lot from observation, reading good books (novels), watching movies or plays, and discussing cases with his peers and teachers. With this training, the neurologist will probably be prepared for comprehensive care: to be the primary physician to diagnose, treat and follow up the patient with neurological disorders. And the neurologist will be irreplaceable.
